# Building a career in inflammatory bowel disease: strategies to identify and cultivate a niche

**DOI:** 10.1093/crocol/otag029

**Published:** 2026-05-04

**Authors:** Alvin T George, Hilary K Michel, Lisa B Malter

**Affiliations:** Inflammatory Bowel Disease Center, The University of Chicago Medicine, Chicago, IL, United States; Nationwide Children’s Hospital and The Ohio State University College of Medicine, Columbus, OH, United States; Division of Gastroenterology, NYU Grossman School of Medicine, New York, NY, United States

Choosing a career in inflammatory bowel disease (IBD) is both exciting and daunting. As a fellow or early-career gastroenterologist, one quickly realizes that IBD encompasses a wide range of career paths. The field sits at the intersection of complex clinical care, rapidly evolving therapeutics, translational science, and patient-centered advocacy. Molding a professional identity in such a dynamic landscape can certainly be mystifying, but the answer sometimes lies in carefully crafting a niche: a focused area of expertise that aligns interests, skills, and institutional opportunities.

Developing a niche is more than an academic exercise. It is an investment in long-term growth. A niche defines professional direction, distinguishes oneself as a valuable resource, and guides scholarly and clinical pursuits. It serves as a “personal mission statement.” Whether a career in academia, private practice, or industry, a well-defined niche offers a unique skillset.

The pathway to discovering a niche begins with curiosity: an interesting patient case, compelling research question or funding opportunity, or systems-level problem that demands attention. At the same time, it is important to keep in mind one’s own strengths and skillset.

Formal training can also play a role. Completing an advanced IBD fellowship allows for focused mentorship and immersion in complex IBD management.^1^ Alternatively, additional training in medical education, epidemiology, health services research, clinical trials, nutrition, medical ethics, public health, or clinical informatics can help develop a complementary skillset that can be distinguishing in the field.

No discussion of career development would be complete without emphasizing mentorship.^2^ Finding the right mentor, or team of mentors, is one of the most impactful steps to take in shaping a niche. A great mentor provides more than advice; they help refine goals, build confidence, and identify opportunities that align with a vision. When seeking mentorship, look for those who take genuine interest in a mentee’s success and whose communication style is complementary. They should challenge a mentee intellectually, advocate for them professionally, and guide them in balancing ambition sustainably.

One of the authors (A.T.G.) spoke with three faculty members from his own institution to better exemplify the unique set of skills and circumstances which may pave a path toward developing a niche.

For Dr. Noa Krugliak Cleveland, Assistant Professor of Medicine and Director of Intestinal Ultrasound at the University of Chicago, the concept of intestinal ultrasound (IUS) was first introduced by her mentor. This led to specialized training overseas, and after identifying the lack of a centralized group to guide IUS in the region, led to the development of the Intestinal Ultrasound Group of the United States and Canada (iUSCAN), a nonprofit organization that has become the leading authority on IUS in North America. She emphasizes the importance of selecting a mentor with a successful track record of mentorship and one who is innovative. At the same time, she also highlights the critical aspect of collaboration in niche development, as “working with others amplifies the reach and impact of one’s work, fosters meaningful professional relationships, and creates opportunities for broader influence, sustained growth, and continued career development.” Her biggest piece of advice: “Remain true to yourself. Pursuing what you love will fuel your passion and persistence, making your work impactful enough to change patient care significantly.”

For Dr. Benjamin McDonald, Assistant Professor of Medicine at the University of Chicago, a team of mentors during his training and prior graduate work in immunology helped him identify open areas of research and hone clinical and translational skills to develop a research plan. This served as a framework in being awarded funding to study perianal Crohn’s disease.

Dr. Tenzin Choden, Assistant Professor of Medicine at the University of Chicago, describes finding her niche of aging and frailty in IBD somewhat unexpectedly. She says, “I was struck by how different my interactions were with older adults compared with younger patients. The medical, social, and functional complexities were distinct, and it quickly became clear that caring for older adults with IBD required an additional layer of thoughtfulness—from treatment tolerance and comorbidities to functional status, frailty, support systems, and long-term goals of care.” She also highlights the significance of mentors who refine curiosity into something real and sustainable, and excellent mentorship involves a careful balance of guidance and fostering independence.

Nevertheless, this guidance in identifying a niche may not require local mentorship. Virtual connections are increasingly accessible, and many structured programs help foster them. The REACH-IBD Career Program through the Crohn’s & Colitis Foundation, for instance, is an excellent way for fellows and early-career faculty to connect with mentors and peers nationwide. Through this program, participants gain exposure to diverse career paths in IBD, receive individualized mentorship, and build a professional community that lasts well beyond training. Moreover, resources such as the American College of Gastroenterology (ACG) Mentoring Program and American Gastroenterological Association (AGA) Career Compass offer a diverse array of mentors along with additional opportunities to support career development.

Sometimes, despite careful planning, a niche will find you. It may start with a single patient whose story provokes a shift in the approach to IBD care or identifies a need to improve the dissemination of knowledge as a clinician educator. It may also arise from a project that grows unexpectedly or a collaboration that opens new doors. These organic moments often define the most rewarding careers, the ones that feel authentic and deeply connected to core values. It does not need to follow anyone else’s particular path but should be something that energizes and motivates.

Once a niche is identified, nurture it through consistent effort and visibility along with institutional support.^3^ Present at conferences, submit work for publication, and participate in collaborative networks. Seek out committees or working groups within professional societies such as the AGA, ACG, or the Crohn’s & Colitis Foundation that align with the area of focus.

Still, staying open to evolution is key. A niche might shift as new therapies, technologies, or patient needs emerge. What begins as a focus on clinical outcomes could evolve into digital health innovation or patient-reported measures. The flexibility to adapt ensures work will remain both relevant and personally fulfilling.

**Figure 1:**
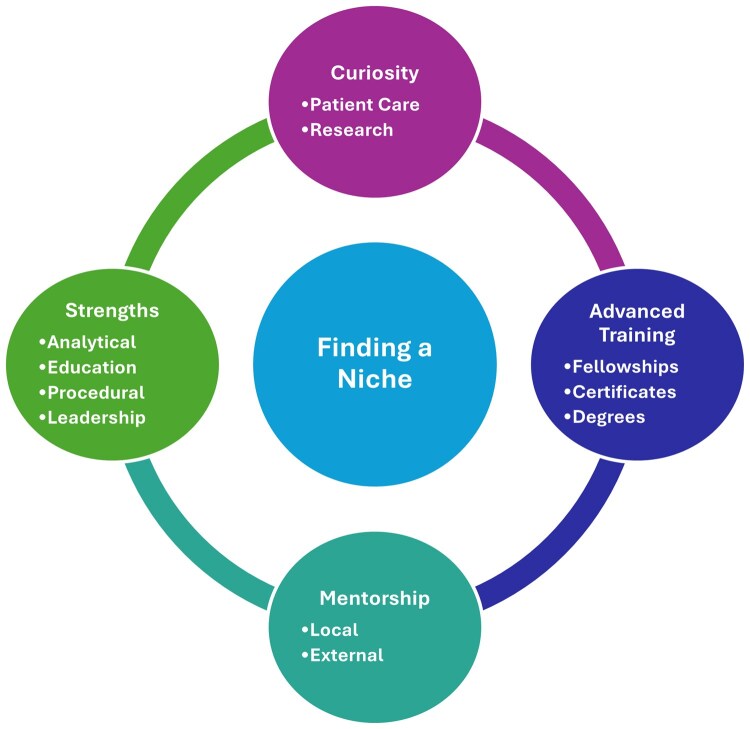
Overall framework for identifying a niche within IBD.

In conclusion, developing a niche in IBD is both a professional and personal journey (Table 1 and Figure 1). It is about aligning what the field needs with what brings joy and purpose. Sometimes a niche is identified specifically and intentionally; other times, it will reveal itself when least expected. Regardless, being surrounded by thoughtful mentors and keeping patients at the center will move things forward. The landscape of IBD care and research is expanding rapidly, and there’s never been a better time to define a place within it.

**Table 1:** Toolkit for identifying a niche within IBD.

Domain	Resources	Description
**Mentorship**	Local institutional leadership REACH-IBD Mentorship Program ACG Mentorship Program AGA Career Compass	Opportunity for structured mentorship and exposure to active IBD research can cultivate a niche Access to learning communities and potential collaborations
**Professional development and exploration**	ACG Institute Leadership YOU AGA Leadership Programs AIBD—Fellows Workshop AIBD—Building an IBD Center Workshop Advanced IBD MILESTONE Program Cornerstones Health Programming	Exposure to diverse topics and provides opportunities to connect individuals with shared interests
**Advanced training, certificate/degree programs**	Advanced IBD fellowship CCF Visiting Fellowship Program Advanced endoscopy Clinical nutrition Epidemiology Medical ethics Public health Medical education Clinical informatics Health services research	Allows targeted skill building and creates value that is complementary. Helps define a niche through unique expertise not widely available.
**Additional educational programs**	ACG Education Universe AGA University IBD Summit NASPGHAN fellowship resources	Educational opportunities which can supplement and refine career interests and providing ongoing education.

Abbreviations: ACG, American College of Gastroenterology; AGA, American Gastroenterological Association; AIBD, Advances in Inflammatory Bowel Diseases; CCF, Crohn’s and Colitis Foundation; IBD, inflammatory bowel disease; NASPGHAN, North American Society for Pediatric Gastroenterology, Hepatology, and Nutrition; REACH-IBD, Rising Educators Academics and Clinicians Helping Inflammatory Bowel Disease.
